# Epithelial-mesenchymal transition in undifferentiated carcinoma of the pancreas with and without osteoclast-like giant cells

**DOI:** 10.1007/s00428-020-02889-3

**Published:** 2020-07-13

**Authors:** Paola Mattiolo, Giulia Fiadone, Gaetano Paolino, Deyali Chatterjee, Riccardo Bernasconi, Paola Piccoli, Claudia Parolini, Mouad El Aidi, Nicola Sperandio, Giuseppe Malleo, Roberto Salvia, Lodewijk A. Brosens, Laura D. Wood, Aldo Scarpa, Rita T. Lawlor, Claudio Luchini

**Affiliations:** 1 Department of Diagnostics and Public Health, Section of Pathology, University and Hospital Trust of Verona, Piazzale Scuro, 10, 37134 Verona, Italy; 2 Department of Pathology and Immunology, Washington University, St. Louis, MO USA; 3 Faculty of Medicine and Pharmacy, University of Rabat, Rabat, Morocco; 4 Department of Surgery, The Pancreas Institute, University and Hospital Trust of Verona, Verona, Italy; 5 Department of Pathology, UMC Utrecht, Utrecht University, Utrecht, the Netherlands; 6 Department of Pathology, Radboud UMC, Nijmegen, the Netherlands; 7 Department of Pathology, Sol Goldman Pancreatic Cancer Research Center, Johns Hopkins University School of Medicine, Baltimore, MD USA; 8 ARC-Net Research Center, University and Hospital Trust of Verona, Verona, Italy

**Keywords:** Pancreatic cancer, EMT, Undifferentiated, Osteoclast, Twist1, Snai2

## Abstract

Undifferentiated carcinoma (UC) and undifferentiated carcinoma with osteoclast-like giant cells (UCOGC) are peculiar variants of pancreatic ductal adenocarcinoma (PDAC), characterized by hypercellularity and absence of glandular patterns. The inflammatory microenvironment is peculiar in UCOGC, since it is dominated by macrophages and osteoclast-like giant cells. However, from a molecular point of view, both UC and UCOGC are very similar to conventional PDAC, sharing alterations of the most common genetic drivers. Clinically, UC usually show a worse prognosis, whereas UCOGC may show a better prognosis if it is not associated with a PDAC component. To highlight potential biological differences between these entities, we investigated the role of the epithelial to mesenchymal transition (EMT) in UC and UCOGC. Specifically, we analyzed the immunohistochemical expression of three well-known EMT markers, namely Twist1, Snai2, and E-cadherin, in 16 cases of UCOGC and 10 cases of UC. We found that EMT is more frequently activated in UC (10/10 cases) than in UCOGC (8/16 cases; *p* = 0.05). Furthermore, in UCOGC, EMT was activated with a higher frequency in cases with an associated PDAC component. Snai2 was the most frequently and strongly expressed marker in both tumor types (10/10 UC, 8/16 UCOGC), and its expression was higher in UC than in UCOGC (mean immunohistochemical score: 4.8 in UC vs. 2.1 in UCOGC, *p* < 0.01). Our results shed new light on the biology of UC and UCOGC: EMT appeared as a more important process in UC, and Snai2 emerged as a central EMT effector in this setting.

**Electronic supplementary material:**

The online version of this article (10.1007/s00428-020-02889-3) contains supplementary material, which is available to authorized users.

## Introduction

Pancreatic cancer is a lethal malignancy with increasing incidence [1–3]. The most common subtype of pancreatic cancer is pancreatic ductal adenocarcinoma (PDAC) [2–4]. The spectrum of PDAC also includes some morphological variants, one of which is the undifferentiated carcinoma (UC), a distinct and hypercellular tumor entity, composed of neoplastic cells without ductal/glandular architecture [3, 4]. Within this PDAC subgroup, there is an even more particular variant, which is the undifferentiated carcinoma with osteoclast-like giant cells (UCOGC). This is morphologically very similar to UC, with the addition of histiocytes and osteoclast-like giant cells intermingled with tumor cells [3, 4].

From the clinical point of view, compared to conventional PDAC, UC usually show a worse prognosis, whereas UCOGC is generally associated to a better prognosis, when not associated with a PDAC component [3, 5, 6]. Some studies have demonstrated that UC and UCOGC have a molecular landscape very similar to PDAC, based on alterations that affect the classic PDAC drivers, including the oncogene *KRAS* and the tumor suppressor genes *TP53*, *SMAD4*, and *CDKN2A* [5, 7, 8]. As such, somatic mutations are unlikely to explain the unique phenotype of UC and UCOGC. A recent study exploring the potential role of inflammatory cells in driving the distinct morphological features of UCOGC, found that the massive recruitment of CD163 positive tumor-associated macrophages and the activation of the PD-1/PD-L1 axis may partly explain its characteristic aspects [9].

A single study reported the involvement of the epithelial to mesenchymal transition (EMT) in the UC variant, but no information is available for UCOGC [10]. EMT is a biological process in which the epithelial elements lose their polarity and cell-to-cell contacts, undergo cytoskeleton remodeling with morphological modifications, and acquire migratory capacity [11]. EMT has recently emerged as a crucial biological mechanism in undifferentiated carcinomas of other organs [12, 13], and in PDAC has been strongly associated to poor prognosis [14–16].

Here we performed an exploratory study aimed at clarifying the potential role played by EMT in UC and UCOGC, through the assessment of the immunohistochemical expression of three well-known EMT-associated factors: E-cadherin, Twist1, and Snai2. Our findings may help to better understand the biology of these particular PDAC subtypes, and generate new insights into the potential role played by EMT in influencing the prognosis of such neoplasms.

## Materials and methods

The cohort of UCOGC and UC analyzed in this study included 16 cases of UCOGC and 10 cases of UC from the pathology archives of Verona University Hospital and The Johns Hopkins University Hospital, which had already been investigated in recent publications by our collaborative research group [5, 9]. As part of such previous studies, this research has been conducted under the approval received by Ethics Committee and Institutional Review Boards of the involved Institutions, in accordance with the Good Practice guidelines, the Declaration of Helsinki and current laws, ethics and regulations.

Four micrometer (μm) formalin-fixed paraffin-embedded sections were immunostained with antibodies for E-cadherin (clone: NCH-38, 1:20 dilution, Dako/USA), Twist1 (Twist2C1a, 1:80, Santacruz/USA), and Snai2 (rabbit, 1:350, Xeptagen/Italy), as previously described [9, 17]. One representative whole-section slide has been used for each case. Briefly, heat-induced antigen retrieval was performed using a heated plate and 0.01 mol/l of citrate buffer, pH 8.9, for 15 min. For Snai2, the antigen-antibody reaction was incubated overnight at 4 °C. Light nuclear counterstaining was performed with hematoxylin. All samples were processed using a sensitive peroxidase-based “Bond polymer Refine” detection system in an automated Bond instrument (Vision-Biosystem, Leica, Milan, Italy). Sections incubated without the primary antibody served as negative controls.

Immunohistochemical staining (IHC) were evaluated separately and blindly by three residents in pathology (P.M., G.F., G.P.) and then reviewed by a pancreatic pathologist (C.L.). Inconsistencies were resolved by consensus at a multi-headed microscope. IHC was considered positive, when nuclei stained for Twist1 and Snai2, and when cell membrane stained for E-cadherin. The overall evaluation was made using a combined quantitative and qualitative score. First, the percentage of positive cells was calculated by assigning a quantitative score, as follows: no positive cells = 0, 1–25% = 1, 26–50% = 2, 51–75% = 3, 76–100% = 4. Then, a qualitative evaluation was performed, assigning a score based on the staining intensity: score 0 = no staining, score 1 = weak staining, score 2 = moderate staining, score 3 = strong staining. Finally, the combined score was calculated by multiplying the quantitative and the qualitative scores, with the final score ranging from 0 to 12. Since there are differences in the expression patterns of the diverse marker, this score can give a more reliable estimation of the actual staining, and could be also more reproducible by future studies. Notably, a positive IHC nuclear staining for Twist1 and/or Snai2, and/or a negative IHC membrane staining for E-Cadherin were interpreted as patterns of EMT activation, as well-known from the literature [11, 14, 17–19]. Due to the intrinsic morphologic features of the tumors investigated in this study (hyerpcellular tumor, presence of inflammatory cells), to avoid IHC misinterpretation and to increase the reliability of our findings, we considered as a pattern of EMT activation only a clear and complete loss of E-cadherin expression in tumor cells.

A further survival analysis was performed to evaluate for any possible prognostic significance of the IHC results, based on the pattern of expression of E-cadherin, Twist1, and Snai2.

## Results

The overall results are summarized in Table 1; quantitative and qualitative IHC scores, that generated the combined IHC score, have been reported for each case in Supplementary Table 1.

**Table 1:** Summary table of the main clinic-pathological features of all investigated cases and of immunohistochemical results

ID	pT	pN	M	TNM	Associated PDAC	VI	PNI	NAT	Twist1	Snai2	E-cad	OS
UCOGC												
1	T2	N1	0	IIB	No	Yes	Yes	No	0	0	1	113
2	T3	N2	0	III	No	Yes	Yes	No	0	0	1	9
3	T2	N0	0	IB	Yes	Yes	Yes	No	0 (0)	0 (2)	1 (0)	28
4	T3	N2	0	III	No	Yes	Yes	No	0	0	1	Na
5	yT2	N0	0	IB	No	Yes	Yes	Yes	1	1	0	Alive (72)
6	T2	N0	0	IB	Yes	Yes	Yes	No	0 (0)	0 (0)	1 (2)	15
7	T3	N1	0	IIB	Yes	Yes	Yes	No	0 (0)	1 (0)	4 (9)	0
8	yT2	N2	0	III	Yes	Yes	Yes	Yes	0 (0)	1 (1)	0 (1)	Alive (22)
9	T3	N1	0	IIB	No	Yes	No	No	0	0	1	Na
10	T3	N2	1	IV	Yes	No	No	No	1 (0)	6 (0)	12 (12)	Alive (9)
11	T3	N0	0	IIA	Yes	Yes	No	No	6 (0)	8 (0)	0 (6)	Alive (10)
12	yT2	N1	0	IIB	Yes	No	Yes	Yes	0 (0)	4 (0)	0 (6)	Alive (19)
13	T3	N0	0	IIA	No	No	No	No	0	0	1	Na
14	T3	N0	0	IIA	No	No	No	No	1	8	0	Alive (12)
15	yT3	N0	0	IIA	No	Yes	Yes	Yes	0	4	1	Na
16	T2	N2	0	III	No	Yes	No	No	0	0	12	Alive (12)
UC												
A1	T2	N1	0	IIB	–	No	Yes	No	0	9	0	Na
A2	T2	N1	0	IIB	–	Yes	Yes	No	0	1	1	Na
A3	yT2	N1	0	IB	–	Yes	Yes	Yes	0	2	0	2
A4	yT3	N0	0	IIA	–	Yes	Yes	Yes	0	6	4	Alive (27)
A5	T1c	N0	0	IA	–	No	No	No	1	6	0	Alive (16)
A6	T2	N2	0	III	–	Yes	Yes	No	0	2	1	11
A7	T2	N1	0	IIB	–	Yes	Yes	No	1	9	0	Alive (10)
A8	T2	N1	0	IIB	–	Yes	Yes	No	1	9	0	2
A9	T2	N1	0	IIB	–	Yes	Yes	No	0	2	4	Na
A10	T1b	N0	0	IA	–	Yes	Yes	No	0	2	0	Alive (267)

*ID*, identification number; *UCOGC*, undifferentiated carcinoma with osteoclast-like giant cells; *UC*, undifferentiated carcinoma; *pT*, pathological tumor stage; *pN*, pathological nodal stage; *TNM*, AJCC 8th edition staging system; *PDAC*, pancreatic ductal adenocarcinoma; *VI*, vascular invasion; *PNI*, perineural invasion; *NAT*, neo-adjuvant therapy; *OS*, overall survival. In the case of UCOGC with an associated PDAC, the results of PDAC are reported in brackets

### UCOGC

The 16 UCOGC cases included 7 pT2 (pathological tumor stage 2) and 9 pT3 cases; the majority of cases (9/16) presented with nodal metastasis at the time of diagnosis, while 7/16 cases were N0. Only one case had a distant metastasis (M1). Seven of the 16 UCOGC were associated with a conventional PDAC component. Most cases had vascular (12/16 cases) and peri-neural invasion (10/16 cases), and 4/16 cases had received neo-adjuvant chemotherapy.

**Fig. 1:**
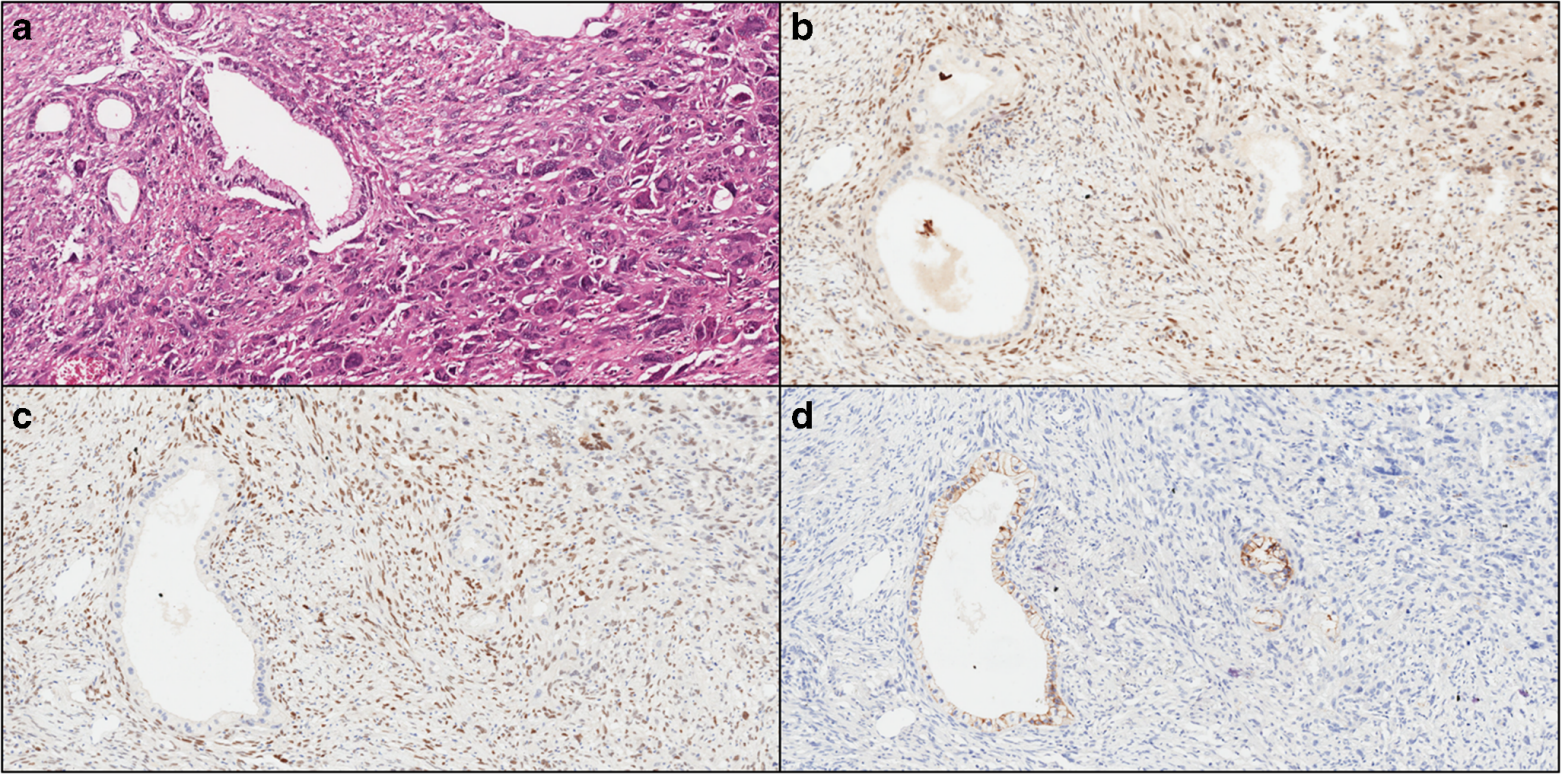
A representative case of undifferentiated carcinoma of the pancreas with osteoclast-like giant cells, with an associated ductal adenocarcinoma, is shown. **a** Hematoxylin-eosin staining reveals an undifferentiated carcinoma with atypical cells and the presence of multinucleated osteoclast-like giant cells; neoplastic glands of the associated ductal adenocarcinoma are also evident (original magnification: × 20). **b** Snai2 is expressed by undifferentiated neoplastic cells, while neoplastic glands are totally negative (original magnification: × 20). **c** Twist1 is expressed by undifferentiated neoplastic cells, while neoplastic glands are totally negative (original magnification: × 20). **d** E-cadherin expression is lost by undifferentiated neoplastic cells, while it is retained by neoplastic glands (original magnification: × 20)

**Fig. 2:**
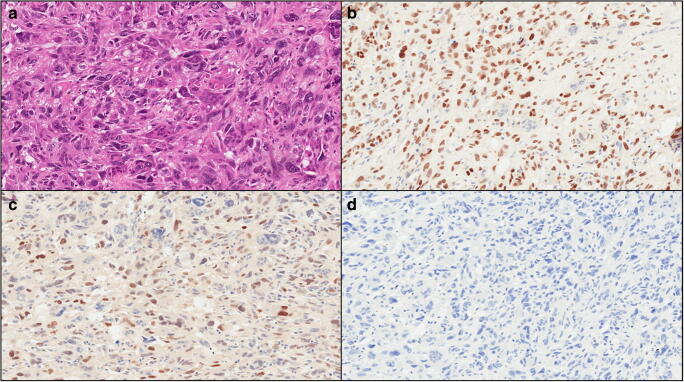
A representative case of undifferentiated carcinoma of the pancreas with osteoclast-like giant cells (without an associated ductal adenocarcinoma) is shown. **a** Hematoxylin-eosin staining reveals an undifferentiated carcinoma with atypical cells and the presence of multinucleated osteoclast-like giant cells (original magnification: × 20). **b** Snai2 is expressed by undifferentiated neoplastic cells, while multinucleated osteoclast-like giant cells are totally negative (original magnification: × 20). **c** Twist1 is expressed by undifferentiated neoplastic cells, multinucleated osteoclast-like giant cells are totally negative (original magnification: × 20). **d** E-cadherin expression is lost by undifferentiated neoplastic cells (original magnification: × 20)

With regard to the expression pattern of EMT markers, for which representative images are provided in Fig. 1 (UCOGC with an associated PDAC component) and Fig. 2 (UCOGC without an associated PDAC component), Twist1 was positive in 4/16 cases, Snai2 in 8/16 cases, and E-cadherin was completely lost in 5/16 cases. Overall, there was evidence of EMT activation in 8/16 cases, based on positive Twist1/Snai2 and/or loss of E-cadherin expression. Snai2 expression had higher scores than those of Twist1, with a mean value of 2.1 vs. 0.6. The associated PDAC components demonstrated expression patterns indicating EMT activation in only 2/7 cases (0/7 Twist expression, 2/7 Snai2 expression, 0/7 E-cadherin loss). However, when a PDAC component was present, the corresponding UCOGC presented an expression pattern indicating EMT activation in the majority of cases (5/7). The higher prevalence of EMT activation in UCOGC with an associated PDAC component, compared to those without associated PDAC, while demonstrating a positive trend, did not however, reach statistical significance (*p* = 0.12, Fisher’s exact test). Among the 9 UCOGC without an associated PDAC component, only 3 cases presented an expression pattern indicating EMT activation: in these cases, Twist1 was positive in 2 cases (both with score 1); Snai2 was present in all cases (with different scores: 1, 4, and 8), and E-cadherin was lost in 2 cases.

All four cases that received neo-adjuvant chemotherapy presented an activated EMT expression pattern (1 case Twist1 positive, all cases Snai2 positive, 3 cases complete E-cadherin loss; Table 1); the prevalence of EMT activation in this particular setting, however, was not statistically significant (4/4 vs. 6/12 that did not receive neoadjuvant chemotherapy; *p* = 0.23, Fisher’s exact test).

The survival analysis based on the expression of Twist1 (positive vs. negative), Snai2 (positive vs. negative), and E-cadherin (maintained vs. lost) did not show any significant result.

### UC

The 10 UC cases included 7 pT2 cases, 2 pT1 cases, and 1 pT3 case; most cases (7/10) presented with nodal metastasis at the time of diagnosis, whereas 3/10 cases were N0. Most cases had vascular (8/10) and peri-neural invasion (9/10 cases), and 2/10 cases received neo-adjuvant chemotherapy.

**Fig. 3:**
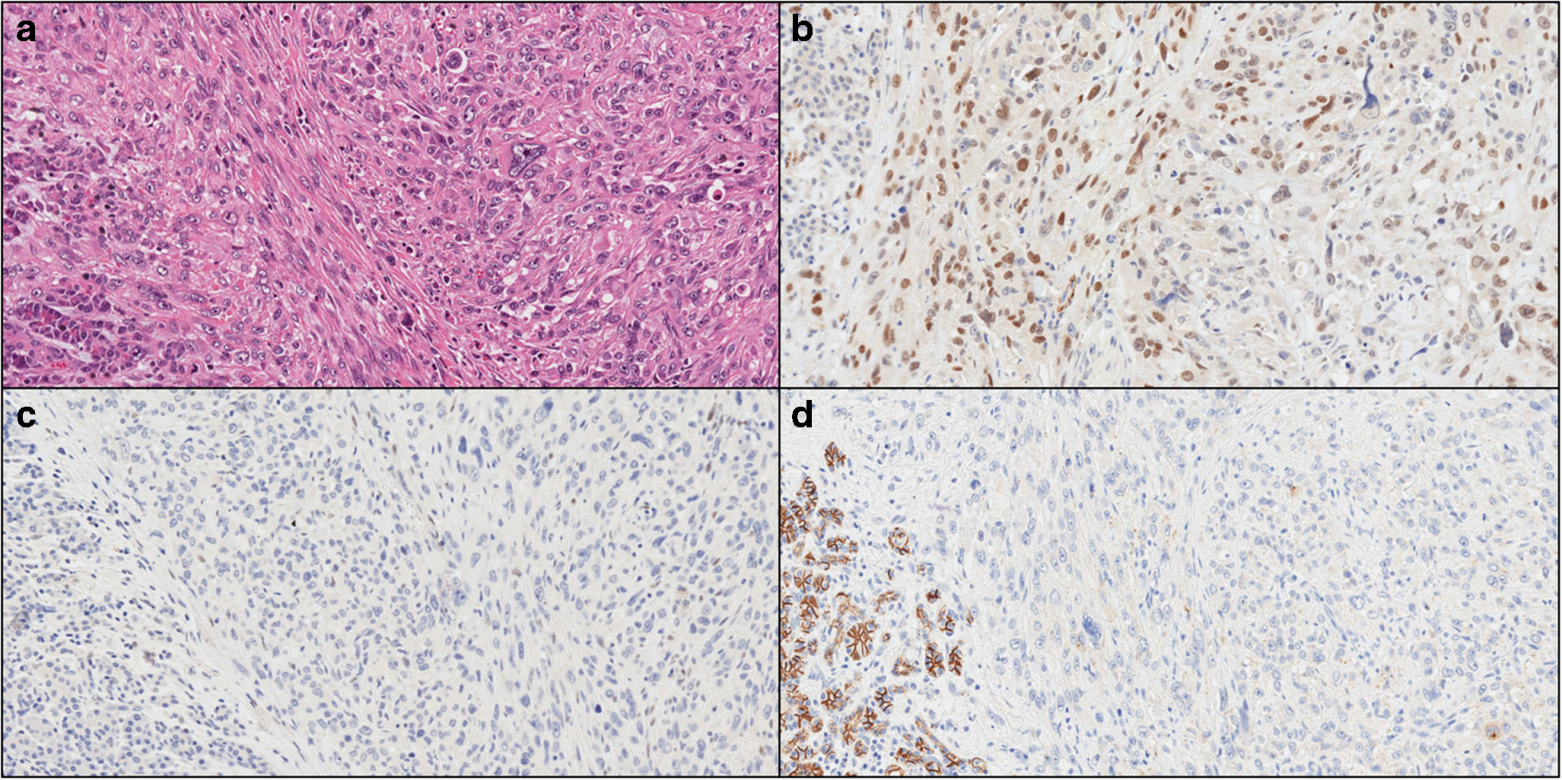
A representative case of undifferentiated carcinoma of the pancreas is shown. **a** Hematoxylin-eosin staining reveals an undifferentiated carcinoma with atypical cells lacking any glandular organization. On the left, there is a thin band of normal pancreatic parenchyma (original magnification: × 20). **b** Snai2 is expressed by most of neoplastic cells and not expressed by normal pancreatic parenchyma (original magnification: × 20). **c** Twist1 is totally negative in neoplastic and normal tissues (original magnification: × 20); **d** E-cadherin expression is lost by neoplastic cells, with the normal pancreatic parenchyma as an internal positive control (original magnification: × 20)

Regarding the expression pattern of EMT markers, of which representative images are provided in Fig. 3, Twist1 was positive in only 3 cases (3/10); Snai2 was expressed in all cases, and E-cadherin was completely lost in 6/10 cases and showed partial or focal loss in all remaining cases. Overall, in all UC cases, there was evidence of an EMT activation based on positive Twist1/Snai2 or loss of expression of E-cadherin.

Similar to UCOGC, survival analysis did not show any significant result in UC cases, based on the expression of Twist1 (positive or negative), Snai2 (positive or negative), and E-cadherin (positive or negative).

### UCOGC vs. UC

**Fig. 4:**
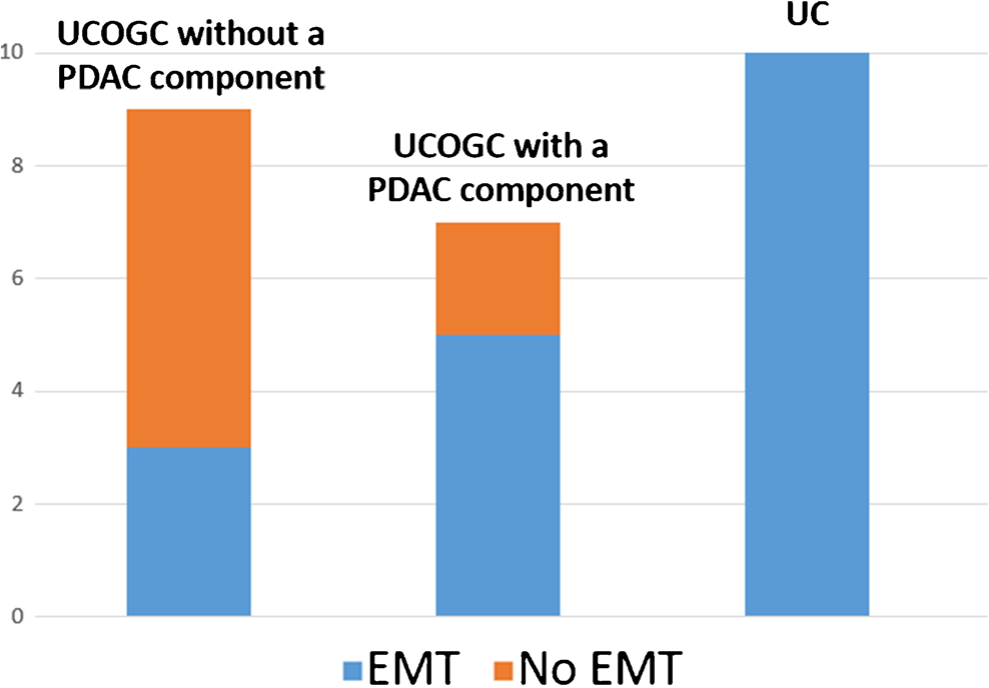
Graphical representation of the prevalence of epithelial-mesenchymal transition (EMT) activation in undifferentiated carcinoma of the pancreas with osteoclast-like giant cells (UCOGC), without and with an associated ductal adenocarcinoma, and in undifferentiated carcinoma of the pancreas without osteoclast-like giant cells (UC)

Comparing the IHC results, EMT was more frequently activated in UC than in UCOGC (10/10 UC vs. 10/16 UCOGC), a difference that reached borderline statistical significance (*p* = 0.05, Fisher’s exact test). The differences in EMT activation between UCOGC, with and without an associated PDAC component, and UC have been graphically summarized in Fig. 4.

While Twist1 expression pattern resulted similar between the two tumor subgroups (5/16 Twist1 positive UCOGC vs. 3/10 positive UC; no statistically significant difference, Fisher’s exact test), the expression of Snai2 was significantly higher in UC than in UCOGC (10/10 positive UC vs. 4/16 positive UCOGC; *p* < 0.01, Fisher’s exact test). The pattern of expression of E-Cadherin did not reach statistically significant difference between UC and UCOGC, but there was a positive trend towards a higher rate of E-cadherin loss in UC.

Twist1 and E-cadherin had a higher mean value of the combined score in UCOCG than UC (for Twist1: 0.6 vs. 0.3, *p* = 0.60, Student’s *t* test; for E-cadherin: 2.2 vs. 1, *p* = 0.35, Student’s *t* test); conversely, for Snai2, it was higher in UC than in UCOGC, and reached a statistically significant value, (4.8 vs. 2.1, *p* = 0.03 Student’s *t* test). This result indicates that EMT is more activated in UC than in UCOGC for both Snai2 and E-cadherin, with Snai2 reaching a statistically significant value, while Twist1 showed an inverse, but not statistically significant, correlation.

## Discussion

In this study, we investigated the immunohistochemical expression of Twist1, Snai2, and E-Cadherin, three well-known markers associated with EMT activation. Overall, we found that EMT is more frequently activated in UC than in UCOGC (*p* = 0.05); in UCOGC, there was evidence of EMT activation in 50% of cases, with a higher frequency in cases that had an associated PDAC component. Snai2 was the most frequently and strongly expressed marker in both tumor types, in addition to being the most important in determining the observed differences between UC and UCOGC, and a higher mean combined score in UC than in UCOGC (*p* = 0.03).

One of the most relevant results of our investigation concerns the different activation-status of the EMT process in UCOGC vs. UC, which was significantly higher in UC. In highlighting the importance of EMT in UC, our results are in line with a previous report, which investigated EMT only in UC, suggesting an important role of this process for this tumor type [10]. Notably, our study also adds novel findings to this initial data, demonstrating that EMT, in contrast to UC, is not so commonly activated in UCOGC. Our results may suggest that EMT cannot be considered as a central process in the biology of UCOGC and cannot explain the unique morphology of this tumor type. Other factors, such as the macrophage infiltration and the overall inflammatory microenvironment may play a significant role in this [9].

Interestingly, in the UCOGC subgroup, cases with associated PDAC component showed more frequent EMT activation in the undifferentiated component, with a positive trend (*p* = 0.12), although not statistically significant. Since UCOGC with associated PDAC has been shown to strongly correlate with poorer prognosis than its “pure” counterpart [5, 6], it may well be that EMT activation plays a role in influencing this difference in clinical outcome. This is in fact the case for conventional PDAC, where activation of the EMT process has been correlated with a poor prognosis [16, 20–23]. Notably, Hong and colleagues reported that, in conventional PDAC, loss of E-cadherin expression and vimentin expression were associated to poor differentiation and shorter survival [22, 23]. We were unable to demonstrate any significant correlation between EMT activation and prognosis in our cohort, but this is most probably due to the relatively small sample size and to the fact that most cases had either no follow-up or only a short follow-up. Therefore, the lack of a clear prognostic value of EMT activation in our UCOGC cohort might be due to this factor rather than due to a true lack of clinical significance. Further studies are needed to explore this potential interesting association, also in the light of the differences that we reported between UCOGC with and without an associated PDAC component.

In our study we also observed non negligible differences between the three investigated markers. E-cadherin is a potentially significant mediator of EMT, and its downregulation usually represents an early event for EMT activation. Several cytokines, triggering downregulation of E-cadherin, subsequently lead to EMT in different cancers, including PDAC [13, 14, 17, 24, 25]. Most of them promote E-cadherin repression through the modulation of a set of pleiotropically acting transcription factors, including members of snail (e.g.,: Snai1, previously known as Snail, and Snai2, previously known as Slug) and basic helix-loop-helix (E47 and Twist1, previously known as Twist) families [26–30]. Beyond their effects on cadherins, they could also potentially activate the transcription of genes characteristic of the mesenchymal state, inducing EMT. Snai2, which is the most important member of the Snail family, can enhance tumor cell proliferation and invasiveness [28, 29], whereas Twist1 can mediate EMT during cancer progression, particularly in the acquisition of metastatic potential [30]. Notably, Snai2 appears the most strongly expressed and the most commonly activated EMT marker in both UC and UCOGC. Although we used a different type of antibody from the previous study on UC, which used the clone C19G7 from Cell Signaling [10], we confirm the importance of this biomarker in this tumor type, also demonstrating here its central role for EMT activation in UCOGC. Notably, Snai2 showed a higher combined score in UC than in UCOGC (*p* < 0.01).

The findings in our cohort also show that Twist1 and Snai2 can be activated independently in both UC and UCOGC, given that in some cases only, one of these markers is expressed in tumor cells. It is also important to note that loss of the E-cadherin staining pattern is not always correlated with Twist1 and/or Snai2 expression. Of course, we only considered complete absence of E-cadherin staining as true lack of expression, and it may be possible that IHC patterns, characterized by a reduction in the intensity of staining and/or a reduction in the percentage of positive cells, might also indicate E-cadherin downregulation. This may explain why in some cases, positivity in Twist1 and/or Snai2 is not coupled with complete E-cadherin loss.

Another interesting result is the more frequent EMT activation in UCOGC after neoadjuvant chemotherapy (NAT), with a positive but not statistically significant trend (*p* = 0.23). Overall, all UC and UCOGC that received NAT in our cohort presented IHC patterns of EMT activation. A recent study focused on NAT-PDAC has recently described frequent expression of EMT-related markers [31]. Our findings are in line with this study and demonstrate that EMT-related markers can be expressed not only in conventional PDAC, but also in UC and UCOGC in a NAT setting.

Our study does have some limitations. First, other potentially important EMT-related markers could be studied to complete the EMT landscape for these tumor types. For example, ZEB1 and ZEB2 have been described as important partners of the three biomarkers investigated in this study [14]. However, after our previous studies on this topic, only limited material was available for research purposes, and we therefore restricted our study to the three important and well-known EMT-related markers. Furthermore, the design of the study was retrospective, and clinical information was incomplete, representing a potential limitation for survival analyses. Finally, the sample size was not very large, although this is of course due to the rarity of such tumors; notably, the present cohort still represents one of the largest cohorts of its kind in the literature.

In conclusion, in this study we have demonstrated that the prevalence of EMT activation was significantly higher in UC than in UCOGC. In both tumor types, Snai2 was the most strongly and commonly expressed marker, with higher IHC scores in UC than in UCOGC. When activated in UCOGC, EMT is more often associated with the presence of an associated PDAC component. Although further studies are needed to confirm our findings of EMT in this setting, This work represents a step forward towards a better comprehension of the biology of UCOGC and UC.

## Electronic supplementary material

ESM 1 (DOCX 13 kb)

## Data Availability

All data and materials as well as software application or custom code support their published claims and comply with field standards.

## References

[1] Rahib L, Smith BD, Aizenberg R, Rosenzweig AB, Fleshman JM, Matrisian LM (2014). Projecting cancer incidence and deaths to 2030: the unexpected burden of thyroid, liver, and pancreas cancers in the United States. Cancer Res.

[2] Kamisawa T, Wood LD, Itoi T, Takaori K (2016). Pancreatic cancer. Lancet.

[3] WHO Editorial Board Members (2019). WHO classification – tumours of the digestive system.

[4] Luchini C, Capelli P, Scarpa A (2016). Pancreatic ductal adenocarcinoma and its variants. Surg Pathol Clin.

[5] Luchini C, Pea A, Lionheart G, Mafficini A, Nottegar A, Veronese N, Chianchiano P, Brosens LAA, Noë M, Offerhaus GJA, Yonescu R, Ning Y, Malleo G, Riva G, Piccoli P, Cataldo I, Capelli P, Zamboni G, Scarpa A, Wood LD (2017). Pancreatic undifferentiated carcinoma with osteoclast-like giant cells is genetically similar to, but clinically distinct from, conventional ductal adenocarcinoma. J Pathol.

[6] Muraki T, Reid MD, Basturk O, Jang KT, Bedolla G, Bagci P, Mittal P, Memis B, Katabi N, Bandyopadhyay S, Sarmiento JM, Krasinskas A, Klimstra DS, Adsay V (2016). Undifferentiated carcinoma with osteoclastic giant cells of the pancreas: clinicopathologic analysis of 38 cases highlights a more protracted clinical course than currently appreciated. Am J Surg Pathol.

[7] Westra WH, Sturm P, Drillenburg P, Choti MA, Klimstra DS, Albores-Saavedra J, Montag A, Offerhaus GJ, Hruban RH (1998). K-ras oncogene mutations in osteoclast-like giant cell tumors of the pancreas and liver: genetic evidence to support origin from the duct epithelium. Am J Surg Pathol.

[8] Li J, Wei T, Zhang J, Wei S, Chen Q, Chen BW, Zhou Y, Wen L, Qin H, Bai X, Liang T (2020) Carcinosarcoma of the pancreas: comprehensive clinicopathological and molecular characterization. HPB (Oxford) 10.1016/j.hpb.2020.01.017 32081541

[9] Luchini C, Cros J, Pea A, Pilati C, Veronese N, Rusev B, Capelli P, Mafficini A, Nottegar A, Brosens LAA, Noë M, Offerhaus GJA, Chianchiano P, Riva G, Piccoli P, Parolini C, Malleo G, Lawlor RT, Corbo V, Sperandio N, Barbareschi M, Fassan M, Cheng L, Wood LD, Scarpa A (2018). PD-1, PD-L1, and CD163 in pancreatic undifferentiated carcinoma with osteoclast-like giant cells: expression patterns and clinical implications. Hum Pathol.

[10] Dongre A, Weinberg RA (2019). New insights into mechanisms of epithelial-mesenchymal transition and implications for cancer. Nat Rev Mol Cell Biol.

[11] Franceschi T, Durieux E, Morel AP, de Saint Hilaire P, Ray-Coquard I, Puisieux A, Devouassoux-Shisheboran M (2019). Role of epithelial-mesenchymal transition factors in the histogenesis of uterine carcinomas. Virchows Arch.

[12] Beuselinck B, Lerut E, Wolter P, Dumez H, Berkers J, Van Poppel H, Joniau S, Oyen R, De Wever L, Strijbos M, Paridaens R, Schöffski P (2014). Sarcomatoid dedifferentiation in metastatic clear cell renal cell carcinoma and outcome on treatment with anti-vascular endothelial growth factor receptor tyrosine kinase inhibitors: a retrospective analysis. Clin Genitourin Cancer.

[13] Galván JA, Zlobec I, Wartenberg M, Lugli A, Gloor B, Perren A, Karamitopoulou E (2015). Expression of E-cadherin repressors SNAIL, ZEB1 and ZEB2 by tumour and stromal cells influences tumour-budding phenotype and suggests heterogeneity of stromal cells in pancreatic cancer. Br J Cancer.

[14] Kohler I, Bronsert P, Timme S, Werner M, Brabletz T, Hopt UT, Schilling O, Bausch D, Keck T, Wellner UF (2015). Detailed analysis of epithelial-mesenchymal transition and tumor budding identifies predictors of long-term survival in pancreatic ductal adenocarcinoma. J Gastroenterol Hepatol.

[15] Lawlor RT, Veronese N, Nottegar A, Malleo G, Smith L, Demurtas J, Cheng L, Wood LD, Silvestris N, Salvia R, Scarpa A, Luchini C (2019). Prognostic role of high-grade tumorbBudding in pancreatic ductal adenocarcinoma: a systematic review and meta-analysis with a focus on epithelial to mesenchymal transition. Cancers (Basel).

[16] Ishida K, Yamashita R, Osakabe M, Uesugi N, Yamada N, Nitta H, Fujishima F, Motoi F, Suzuki H, Shimamura H, Noda Y, Sawai T, Unno M, Sasano H, Sasaki A, Sugai T (2019). Expression of epithelial-mesenchymal transition proteins in pancreatic anaplastic (undifferentiated) carcinoma. Pancreas.

[17] Luchini C, Parcesepe P, Mafficini A, Nottegar A, Parolini C, Veronese N, Remo A, Manfrin E (2015). Specific expression patterns of epithelial to mesenchymal transition factors in gestational molar disease. Placenta.

[18] Loh CY, Chai JY, Tang TF, Wong WF, Sethi G, Shanmugam MK, Chong PP, Looi CY (2019). The E-cadherin and N-cadherin switch in epithelial-to-mesenchymal transition: signaling, therapeutic implications, and challenges. Cells.

[19] Zhu QQ, Ma C, Wang Q, Song Y, Lv T (2016). The role of TWIST1 in epithelial-mesenchymal transition and cancers. Tumour Biol.

[20] Krebs AM, Mitschke J, Lasierra Losada M, Schmalhofer O, Boerries M, Busch H, Boettcher M, Mougiakakos D, Reichardt W, Bronsert P, Brunton VG, Pilarsky C, Winkler TH, Brabletz S, Stemmler MP, Brabletz T (2017). The EMT-activator Zeb1 is a key factor for cell plasticity and promotes metastasis in pancreatic cancer. Nat Cell Biol.

[21] Chong Y, Thakur N, Paik KY, Lee EJ, Kang CS (2020). Prognostic significance of stem cell/ epithelial-mesenchymal transition markers in periampullary/pancreatic cancers: FGFR1 is a promising prognostic marker. BMC Cancer.

[22] Hong SM, Li A, Olino K, Wolfgang CL, Herman JM, Schulick RD, Iacobuzio-Donahue C, Hruban RH, Goggins M (2011). Loss of E-cadherin expression and outcome among patients with resectable pancreatic adenocarcinomas. Mod Pathol.

[23] Handra-Luca A, Hong SM, Walter K, Wolfgang C, Hruban R, Goggins M (2011). Tumour epithelial vimentin expression and outcome of pancreatic ductal adenocarcinomas. Br J Cancer.

[24] Bhowmick NA, Neilson EG, Moses HL (2004). Stromal fibroblasts in cancer initiation and progression. Nature.

[25] Moreno-Bueno G, Portillo F, Cano A (2008). Transcriptional regulation of cell polarity in EMT and cancer. Oncogene.

[26] Moreno-Bueno G, Cubillo E, Sarrió D, Peinado H, Rodríguez-Pinilla SM, Villa S, Bolós V, Jordá M, Fabra A, Portillo F, Palacios J, Cano A (2006). Genetic profiling of epithelial cells expressing E-cadherin repressors reveals a distinct role for snail, slug, and E47 factors in epithelial-mesenchymal transition. Cancer Res.

[27] Shah PP, Kakar SS (2011). Pituitary tumor transforming gene induces epithelial to mesenchymal transition by regulation of twist, snail, slug, and E-cadherin. Cancer Lett.

[28] Batlle E, Sancho E, Francí C, Domínguez D, Monfar M, Baulida J, García de Herreros A (2000). The transcription factor snail is a repressor of E-cadherin gene expression in epithelial tumor cells. Nat Cell Biol.

[29] Cobaleda C, Pérez-Caro M, Vicente-Dueñas C, Sánchez-García I (2007). Function of the zinc-finger transcription factor SNAI2 in cancer and development. Annu Rev Genet.

[30] Yang J, Mani SA, Donaher JL, Ramaswamy S, Itzykson RA, Come C, Savagner P, Gitelman I, Richardson A, Weinberg RA (2004). Twist, a master regulator of morphogenesis, plays an essential role in tumor metastasis. Cell.

[31] Wang M, Estrella JS, Katz MH, Kim M, Rashid A, Lee JE, Maitra A, Wistuba II, Wolff RA, Varadhachary GR, Wang H (2019). Expression of epithelial-mesenchymal transition markers in treated pancreatic ductal adenocarcinoma. Pancreas.

