# Supplementary material for: Epithelial-mesenchymal transition in undifferentiated carcinoma of the pancreas with and without osteoclast-like giant cells
Source: Virchows Arch. 2020 Jul 13;478(2):319–26. doi: 10.1007/s00428-020-02889-3 (PMC7969490; doi:10.1007/s00428-020-02889-3)
Supplement: Supplementary file 1 — (DOCX 13 kb) [file 428_2020_2889_MOESM1_ESM.docx]

**Supplementary Table 1**. Summary table reporting for each case all values of quantitative and qualitative scores for obtaining the final combined score.

| **ID** | | **Twist1** | | **Snai2** | | **E-Cad** |
| --- | --- | --- | --- | --- | --- | --- |
| **UCOGC** |  | |  | |  |  |
| 1 | | 0 | | 0 | | 1x1=1 |
| 2 | | 0 | | 0 | | 1x1=1 |
| 3 | | 0 (0) | | 0 (2x1=2) | | 1x1=1 (0) |
| 4 | | 0 | | 0 | | 1x1=1 |
| 5 | | 1x1=1 | | 1x1=1 | | 0 |
| 6 | | 0 (0) | | 0 (0) | | 1x1=1 (1x2=2) |
| 7 | | 0 (0) | | 1x1=1 (0) | | 2x2=4 (3x3=9) |
| 8 | | 0 (0) | | 1x1=1 (1x1=1) | | 0 (1x1=1) |
| 9 | | 0 | | 0 | | 1x1=1 |
| 10 | | 1x1=1 (0) | | 3x2=6 (0) | | 4x3=12 (4x3=12) |
| 11 | | 2x3=6 (0) | | 4x2=8 (0) | | 0 (3x2=6) |
| 12 | | 0 (0) | | 2x2=4 (0) | | 0 (2x3=6) |
| 13 | | 0 | | 0 | | 1x1=1 |
| 14 | | 1x1=1 | | 4x2=8 | | 0 |
| 15 | | 0 | | 2x2=4 | | 1x1=1 |
| 16 | | 0 | | 0 | | 4x3=12 |
| **UC** |  | |  | |  |  |
| A1 | | 0 | | 3x3=9 | | 0 |
| A2 | | 0 | | 1x1=1 | | 1x1=1 |
| A3 | | 0 | | 2x1=2 | | 0 |
| A4 | | 0 | | 3x2=6 | | 2x2=4 |
| A5 | | 1x1=1 | | 3x2=6 | | 0 |
| A6 | | 0 | | 2x1=2 | | 1x1=1 |
| A7 | | 1x1=1 | | 3x3=9 | | 0 |
| A8 | | 1x1=1 | | 3x3=9 | | 0 |
| A9 | | 0 | | 1x2=2 | | 2x2=4 |
| A10 | | 0 | | 2x1=2 | | 0 |

**Abbreviations**: ID: identification number; UCOGC: undifferentiated carcinoma with osteoclast-like giant cells; UC: undifferentiated carcinoma.

**Notes**: in the case of UCOGC with an associated PDAC, the results of PDAC are reported in brackets.
